# *Burkholderia pseudomallei* Bacteria in Ornamental Fish Tanks, Vientiane, Laos, 2023

**DOI:** 10.3201/eid3003.231674

**Published:** 2024-03

**Authors:** Tim Venkatesan, Vannavong Siritana, Joy Silisouk, Tamalee Roberts, Matthew T. Robinson, David A.B. Dance

**Affiliations:** Lao-Oxford-Mahosot Hospital-Wellcome Trust Research Unit, Vientiane, Laos (T. Venkatesan, V. Siritana, J. Silisouk, T. Roberts, M.T. Robinson, D.A.B. Dance);; London School of Hygiene and Tropical Medicine, London, UK (T. Venkatesan, D.A.B. Dance);; University of Oxford Centre for Tropical Medicine and Global Health, Oxford, UK (T. Roberts, M.T. Robinson, D.A.B. Dance)

**Keywords:** *Burkholderia pseudomallei*, melioidosis, fish tank, Vientiane, Laos, bacteria, bacterial infections

## Abstract

In 2019, a melioidosis case in Maryland, USA, was shown to have been acquired from an ornamental fish tank contaminated with *Burkholderia pseudomallei* bacteria, likely derived from Southeast Asia. We investigated the presence of *B. pseudomallei* in ornamental fish tanks in the endemic area of Vientiane, Laos.

*Burkholderia pseudomallei* is a saprophytic gram-negative bacillus that resides in the soil and surface water of many tropical and subtropical environments (*1*). This bacterium causes the potentially life-threatening infection melioidosis, a major cause of death in endemic areas (*1*).

In 2019, a 56-year-old woman from Maryland, USA, was hospitalized with melioidosis despite having no travel history to a *B. pseudomallei*–endemic region. She was infected with a *B. pseudomallei* isolate found within a recently purchased ornamental fish tank (*2*). Whole-genome sequencing demonstrated genome clustering associated with Southeast Asia. An earlier study had also detected *B. pseudomallei* bacteria in water used to import tropical fish from Singapore to France (*3*). A large overlap exists between *B. pseudomallei* bacteria endemicity and sources of ornamental fish exportation, and Southeast Asia accounts for 57% of global trade (*4*). 

We sampled retail and residential fish tanks in Vientiane Capital, Laos, where *B. pseudomallei* bacteria has been shown to be widespread (*5*,*6*). We defined a fish tank as a container (glass, plastic, or ceramic) with water containing ornamental fish. Samples were collected during the Laos rainy season (June–July), when melioidosis incidence is highest (*7*). Each site completed a questionnaire detailing tank water sources and maintenance procedures.

Sampling methods mirrored those used by the investigational team from the Maryland case (*2*), alongside established methods for environmental sampling of *B. pseudomallei* bacteria in Laos (*6*). From each tank, we took a 1-L water sample, 10 g of sediment, and 2 swab samples (Medical Wire & Equipment, https://www.mwe.co.uk) of biofilm. We vacuumed 500-mL water samples that had been filtered in succession through 5 µm– and 0.2 µm–pore-sized cellulose acetate filters (Sartorius Stedim Biotech, https://www.sartorius.com) to capture suspended particulates and planktonic bacteria. We placed the water filters, the sediment, and swab tips directly in *B. pseudomallei*–selective broth containing colistin (50 mg/mL) and incubated them aerobically at 37°C for 48 hours and 168 hours before culture and molecular detection. We then subcultured 10 µL of enriched sample on Ashdown agar containing gentamicin (8 mg/L). We tested any colony with an appearance consistent with *B. pseudomallei* bacteria by using both *B. pseudomallei*–specific latex agglutination (Mahidol University Faculty of Tropical Medicine, https://www.tm.mahidol.ac.th) and Vitek MS matrix-assisted laser desorption/ionization time-of-flight mass spectrometry (bioMérieux, https://www.biomerieux.com). We conducted molecular detection by using real-time quantitative PCR (qPCR) after 7 days of enrichment in *B. pseudomallei*–selective broth. We performed DNA extraction by using a GeneJET Genomic DNA Purification Kit (ThermoFisher Scientific, https://www.thermofisher.com). The qPCR targeted the *B. pseudomallei* type 3 secretion system using a protocol based on a previously published methodology (*8*). To control for the presence of inhibitors, we used a parallel *Orientia tsutsugamushi* bacteria inhibition control PCR to check delay in amplification of a 47-kDa *O. tsutsugamushi* gene plasmid in the presence of each sample. We processed 2 positive controls using tank water samples we inoculated with 3 and 30 CFU/mL using the same methods. We isolated *B. pseudomallei* bacteria on culture and detected it by qPCR in both cases.

We sampled a total 111 tanks from 14 sites, including 82 tanks from 6 fish retailers and 29 tanks from 8 residents. Eleven (9.9%) tanks were kept outside, 39 (35.1%) were kept outside under cover, and 60 (54.1%) were kept inside. All sites used tap water as the primary water source without the addition of disinfectants, except 1 that used rainwater. We detected *B. pseudomallei* bacteria by qPCR only within a single covered outdoor retailer tank water sample, a finding we confirmed on repeat qPCR testing (cycle threshold value 34.9). The absence of positive culture and the high qPCR cycle threshold value suggested that a low concentration of *B. pseudomallei* bacteria was present in the sample (<1 CFU/500 mL).

Our study has confirmed that *B. pseudomallei* bacteria can contaminate ornamental fish tanks in an endemic area, yet its presence is not widespread in Vientiane Capital, Laos. Our findings probably underestimate the presence of *B. pseudomallei* bacteria, given the limitations in the sensitivity of environmental sampling methods, which have not been optimized for ornamental fish tanks. Because untreated tap water was the primary water source for tanks, the absence of *B. pseudomallei* bacteria suggests it is not widely present in tap water in Vientiane Capital. To our knowledge, no formal analysis of tap water samples in Vientiane has been performed; however, 2 studies undertaken in rural Thailand found *B. pseudomallei* bacteria present within some tap water samples (*9*,*10*). Our positive finding on qPCR does not prove the existence of viable organisms, but it is a possibility. Further studies are needed to investigate possible contamination of tanks in other regions and to determine the risks this might imply for the international ornamental fish trade. We suggest that susceptible persons having contact with fish tanks should take precautions and wear protective gloves while minimizing contact with fish tanks.
